# Preparation of Intercalated Organic Montmorillonite DOPO-MMT by Melting Method and Its Effect on Flame Retardancy to Epoxy Resin

**DOI:** 10.3390/polym13203496

**Published:** 2021-10-12

**Authors:** Junming Geng, Jianyu Qin, Jiyu He

**Affiliations:** 1 School of Materials Science and Engineering, Beijing Institute of Technology, Beijing 100081, China; sscgjm@bit.edu.cn; 2 Beijing System Design Institute of Electro-Mechanic Engineering, Beijing 100854, China; qinjianyu2016@163.com

**Keywords:** 9,10-dihydro-9-oxa-10-phosphaphenanthrene-10-oxide, intercalated organic montmorillonite, epoxy resin, flame retardant

## Abstract

An intercalated organic montmorillonite DOPO-MMT was prepared through the melting method using 9,10-dihydro-9-oxa-10-phosphaphenanthrene-10-oxide (DOPO) as a modifier. Epoxy resin (EP) composites were prepared with DOPO-MMT, DOPO, MMT, and the physical mixtures of DOPO+MMT as flame retardants. The microstructure of the flame retardants and EP samples were characterized by X-ray diffraction (XRD), scanning electron microscopy (SEM), and transmission electron microscopy (TEM). The flame retardant properties, thermal stability, and residual char structure of the EPs were studied by the limited oxygen index (LOI) test, the UL-94 vertical burning test, thermogravimetric analysis (TGA), the differential scanning calorimeter (DSC) test, the cone calorimeter (CONE) test as well as other characterization methods. The results showed that the intercalated organic montmorillonite DOPO-MMT can be successfully prepared by the melting method and that the MMT is evenly dispersed in the EP/DOPO-MMT composite in the form of nanosheets. The EP/DOPO-MMT nanocomposites showed the optimal flame retardancy (LOI, UL-94, PHRR, etc.) among the EPs with DOPO, MMT, and the physical mixture of DOPO+MMT. The flame-retardant grade of the material reached V-0.

## 1. Introduction

MMT is halogen-free, non-toxic, low-cost, and an attractive inorganic nanoscale particle for flame retardant polymer materials [1]. The well-dispersed montmorillonite nanosheets in the resin can be used as a good barrier to inhibit the heat transfer and escape of volatile components in the combustion process and to promote the resin to form a stronger char layer in the condensed phase. Moreover, the char layer is an effective protective barrier to slow down the further degradation of the resin matrix [2,3,4]. However, in resin materials in general, the surface of unmodified montmorillonite in its original state presents a completely hydrophilic inorganic phase, and it is less likely to be intercalated or exfoliated directly in the hydrophobic and internally incompatible resin matrix, which usually exists at the micron size [5,6,7,8,9,10,11].

In order to prepare nano-dispersed montmorillonite nanocomposites, it is necessary to organically modify the natural montmorillonite to enhance its compatibility with the resin matrix. It is one of the most widely used methods to improve the compatibility between montmorillonite and the polymer using cationic quaternary alkyl ammonium or alkyl phosphine to exchange the original metal cations such as Na^+^ or Ca^2+^ between the layers, which can expand the spacing between the layers of montmorillonite, realize the organic surface of the lamellar, and improve the affinity with the organic matrix [12,13,14,15]. However, the amino group in the cationic organic modifier affects the thermal stability of cationic organic montmorillonite (OMMT), which is difficult to meet the thermal stability and flame-retardant performance requirements of polymer/montmorillonite composites. Therefore, in order to improve the thermal stability and surface properties of organic modifiers, experts and scholars in this field prepared anionic OMMT [16], nonionic OMMT [17,18], anionic–nonionic OMMT [19,20], anionic–cationic OMMT [21,22], and cationic–nonionic OMMT [23,24], using anionic surfactants (sodium dodecyl sulfonate and sodium stearate), non-ionic surfactants, and two kinds of combined surfactants (anionic+nonionic surfactants, anionic+quaternary ammonium surfactant, nonionic+quaternary ammonium surfactant) as modifiers.

However, all of the organically modified montmorillonites prepared with surfactants have many problems in terms of improving the flame retardancy of polymers. On the one hand, the organic modifier that is used does not have flame-retardant properties and is flammable itself, so the flame-retardant efficiency of this kind of organically modified montmorillonite is low. On the other hand, the process of preparing organically modified montmorillonite by means of the cation exchange method is generally conducted in aqueous solution, in which it is difficult to avoid producing more polluting wastewater.

In order to solve the above problems, the modification of montmorillonite using flame retardants directly has been explored. Phosphorus flame retardants have better light stability than most halogen flame retardants, and because of their greater biodegradation possibility, they have been widely concerned and studied at home and abroad [25,26,27]. In our laboratory, a variety of phosphorus-containing flame retardants—MMT nanocomposites were prepared using phosphorus-containing flame retardants as modifiers, and good flame retardants were obtained [28,29,30,31]. Yi prepared intercalated ammonium polyphosphate (APP)-MMT nanocompounds through a reaction of diammonium hydrogen phosphate, phosphorous pentoxide, and urea with the addition of MMT in a well-covered kneading reactor. The nano-MMT layers enhanced the thermal stability of APP at a high temperature, and the APP-MMT nanocompounds improved the thermal stability and the flame retardancy of the PP composites. However, the process was cumbersome, and toxic ammonia was also introduced into the reaction process. The intercalated DOPO-MMT was prepared by He with DOPO and MMT (mass ratio 5:1) in an ethanol solution. When 6 wt % DOPO-MMT was added into the epoxy composites, the LOI of EP/DOPO-MMT (33.4%) was significantly improved compared to pure EP (23.0% and the flame retardant rating reached V-0. In this solution preparation, the ethanol solvent needed to be recovered, and the intercalation efficiency was low. In view of the above problems, such as the low intercalation efficiency, high cost, and cumbersome process, it is very necessary to explore a principle and method that is suitable for the direct modification of montmorillonite by flame retardants and to prepare organic montmorillonite with a synergistic flame-retardant effect.

In this article, an intercalated DOPO-MMT was directly prepared using the melt intercalation method with a 2:1 mass ratio of DOPO to MMT. Moreover, the intercalated DOPO-MMT was compared with DOPO, MMT, and physically mixed DOPO+MMT by means of XRD, SEM, TEM, the LOI test, the UL-94 vertical burning test, TGA, DSC, and the CONE test. Furthermore, its structure, morphology, thermal stability, and flame-retardant properties in EP resin were studied to evaluate the contribution of DOPO-MMT nanocomposites to the flame retardancy and thermal stability of epoxy resin. Finally, an intercalated organic montmorillonite DOPO-MMT with excellent flame retardancy, simple preparation that was environmentally friendly and low cost was obtained.

## 2. Materials and Methods

### 2.1. Materials

**Figure 1:**
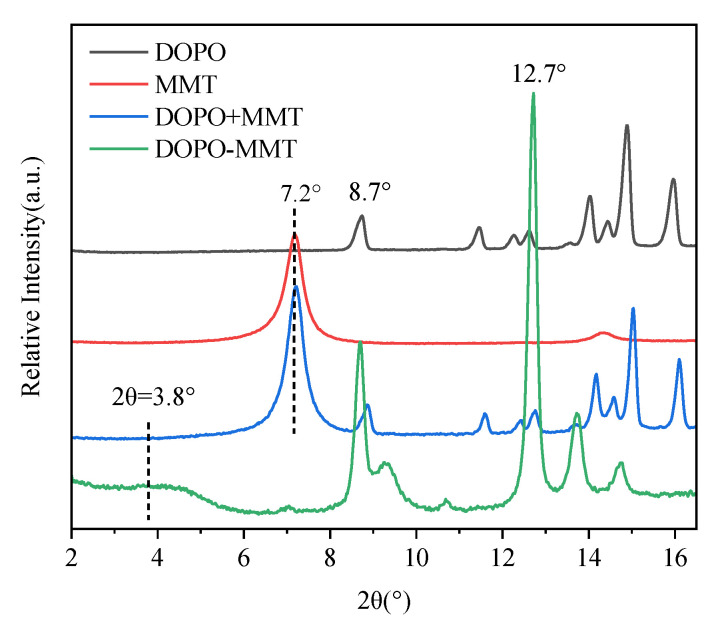
XRD patterns of four flame retardants.

Na-montmorillonite (MMT) was purchased from the American Nanocor Corporation (Hoffman Estates, IL, USA). The compound 9, 10-dihydrogen, 9-oxa, 10-phosphorus, phenanthrene, 10-oxide (DOPO, analytically pure, the structural formula is shown in Figure 1) was purchased from Shanghai Youdi Chemicals Co. (Shanghai, China). Diglycidyl ether of bisphenol A (DGEBA, E-44) was purchased from Feicheng Deyuan Chemicals Co., Ltd. (Taian, China). A curing agent, 4,4-diaminodiphenylsulphone (DDS), was purchased from the Tianjin Guangfu Fine Chemical Research Institute (Tianjin, China).

### 2.2. Preparation of Intercalated DOPO-MMT

The DOPO-MMT nanocomposites were prepared with the 2:1 mass ratio of DOPO to MMT. An amount of 50 g DOPO was melted in a three-tip flask at 140 °C and was stirred for 5 min. An amount of 25 g MMT was subsequently added to the flask. Then, the mixture was stirred continuously at 140 °C and at 300 RPM for 8 h, was poured out, cooled to room temperature, and was then ground through a 200-mesh screen. The final mixture was called DOPO-MMT.

### 2.3. Preparation of Physical Mixed Sample DOPO+MMT

The DOPO and MMT (mass ratio: 2:1), which had been ground through the 200-mesh screen, were simply mixed for 10 min. The physical mixture of DOPO and MMT was obtained as DOPO+MMT, which was used as the comparison sample for the DOPO-MMT nanocomposite.

### 2.4. Preparation of Epoxy Resin Composites

Epoxy resin composites were prepared using a thermal curing process. First, the flame retardants, such as the MMT, DOPO, physical mixture of DOPO+MMT, and the DOPO-MMT nanocomposite were dispersed in DGEBA by mechanical stirring at 140 °C for 2 h. Then, the curing agent DDS was added to DGEBA based on a 10:3 (*w*/*w*) ratio of DGEBA to DDS, and the mixture was continuously stirred at 140 °C for 0.5 h. Finally, the mixture was poured into a PTFE mold with a certain shape and was cured horizontally in a drying oven at 180 °C for 4 h. The final epoxy resin composites were called EP/MMT, EP/DOPO, EP/DOPO+MMT, and EP/ DOPO-MMT. The addition of MMT, DOPO, DOPO+MMT, and DOPO-MMT is 6% of the total mass of the epoxy resin.

### 2.5. Instrumentation and Methods for Testing Characterization

#### 2.5.1. XRD Characterization

The X-ray diffraction (XRD) patterns of the samples were obtained using Cu-Kα radiation (λ = 1.54178 Å) on a MiniFlex 600X-ray diffractometer (Rigaku Corporation, Tokyo, Japan) operating at 40 kV and 15 mA (2θ range from 2° to 20° with a step size of 0.02°). Repeatability was tested by measuring two samples.

#### 2.5.2. SEM Characterization

The microstructures of the samples were observed by means of an FEI Quanta 250 field-emission scanning electron microscope (FEI company, Hillsboro, Oregon, USA). The resin sample was prepared using a low-temperature fracture method under liquid nitrogen cooling and was sprayed with gold before testing. The distribution of Si was determined by means of an energy-dispersive X-ray spectrometer (EDX, EX-350, FEI company) coupled with the SEM. A transmission electron microscope (Fei-Tecnai G2-F30, FEI company) was employed to observe the morphologies of the samples. The acceleration voltage was 300 kV. Epoxy resin samples were sliced into ultrathin sections before testing.

#### 2.5.3. LOI Analysis

The minimum oxygen concentration required for the combustion of a self-supporting candlelight plastic sample was measured in accordance with standard GB/T 2406-93 with a sample size of 120 mm × 6.5 mm × 3 mm. The FTAII oxygen index meter was purchased from Rheometric Scientific LTD., Leatherhead, UK.

#### 2.5.4. UL-94 Vertical Combustion Analysis

Vertical burning tests were performed based on an ASTM D3801-2010 CZF-5A instrument (Jiangning Analytical Instrument Co., LTD., Nanjing, China) with samples of the dimensions 125.0 mm × 13.0 mm × 3.2 mm. In this test, the burning grade of a material is classified as V-0, V-1, V-2, or no grade (NR), depending on its behavior (with or without droplet, burning time after each removal of ignition source).

#### 2.5.5. TGA Characterization

TGA was performed with a Netzsch 209 F1 thermal analyzer (Netzsch, Selb, Germany), with the measurements being conducted in a nitrogen and air atmosphere at a heating rate of 10 °C/min from 40~900 °C. A sample of 2~3 mg was used for each measurement. The TGA results were typically reproducible to within ±1%, and the reported data are the average values from three measurements.

#### 2.5.6. DSC Characterization

A Netzsch DSC 214 instrument (Netzsch, Selb, Germany) under a nitrogen gas flow (60 mL/min) was used to investigate the endothermic process. The samples were first heated from 40 to 240 °C and then cooled to 40 °C and reheated to 240 °C at a heating rate of 10 °C/min. The second heating thermograms were recorded. Typical results from DSC were reproducible within ±1%, and the reported results are the average of three measurements.

#### 2.5.7. CONE Characterization

Cone calorimeter measurements were performed using Fire Testing Technology Combustion equipment (FTT0007, East Grinstead, UK) with a conical thermal radiator with truncated top in accordance with ISO 5660. Without mesh coverage, the specimen (100 mm × 100 mm × 3 mm) was exposed horizontally to a constant radiation flux of 50 kW/m^2^, and the heat release value and mass reduction were continuously recorded during combustion. Typical results from the cone calorimeter tests were reproducible to within ±10%, and the reported data are the averages from three measurements. Samples from the LOI test, UL-94 Vertical combustion test, and CONE were pretreated at 23 °C and at 50% relative humidity for 24 h before testing.

## 3. Results

### 3.1. XRD Analysis of Four Flame Retardants

The prepared DOPO-MMT nanocomposite, and the physical mixture of the DOPO+MMT, MMT and DOPO were characterized by XRD, and the results are shown in Figure 1.

As it can be seen from Figure 1, the diffraction characteristic peak of the Na-montmorillonite is located at 7.2°, corresponding to the layer spacing(d-spacing) of 1.2 nm according to the Bragg equation (2dsinθ = nλ). The characteristic peaks of the DOPO samples appeared at 8.7° and 12.7°. The diffraction peak position of the physical mixture of DOPO+MMT is a simple superposition of the characteristic peak position of DOPO and MMT. This indicates that simple physical mixing maintains the initial state of MMT, and no intercalation reaction occurs. The DOPO-MMT prepared by the melting method shows a lower diffraction peak of montmorillonite at 3.8°, with the exception of the characteristic peak of DOPO. The d-spacing of DOPO-MMT can be calculated as 2.3 nm. This result indicates that there was a large amount of DOPO molecules inserted between the layers of the DOPO-MMT complex, and the intercalated structure of the organic montmorillonite DOPO-MMT was obtained as well.

DOPO is able to intercalate MMT because the surface of montmorillonite is a silicon-oxygen tetrahedron with a lot of oxygen atoms. At the same time, the lamellar edge of montmorillonite has a large number of strongly acidic bridging hydroxyl and weakly acidic silicon hydroxyl. When DOPO exists in the molecular state in a molten state, the active hydrogen in the DOPO molecule will form hydrogen bonds with the oxygen on the surface of the montmorillonite and the hydroxyl group at the end position, allowing it to adsorb on the inner and outer surfaces and the end positions of the montmorillonite. Intercalated organic montmorillonite DOPO-MMT was obtained by the diffusion and adsorption of the DOPO molecules into the interlayer of the montmorillonite.

### 3.2. XRD Analysis of Epoxy Resin Composite

**Figure 2:**
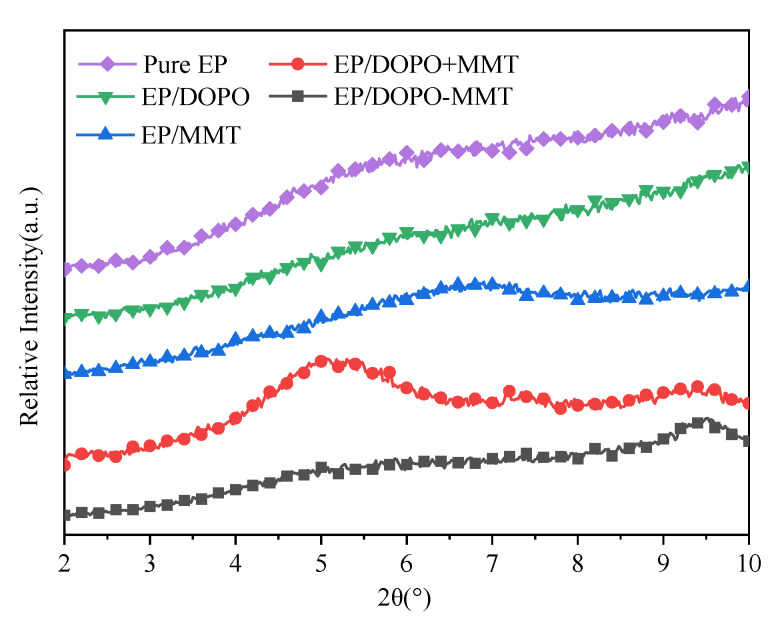
XRD pattern of EPs.

The composites EP/MMT, EP/DOPO, EP/DOPO+MMT, and DOPO-MMT were prepared by the combination of MMT, DOPO, DOPO+MMT, and DOPO-MMT with EP, respectively. The prepared composites were characterized by XRD, and the results are shown in Figure 2.

The results in Figure 2 show that there is no obvious diffraction peak between 2° and 10° for both Pure EP and EP/DOPO, which indicates that DOPO was fully reacted/dissolved in EP and that there was no crystal formation. As it can be seen from Figure 2, EP/MMT has a characteristic peak similar to MMT at 6.9°. This result indicates that MMT exists in a layered structure after being formed as a composite with EP. Figure 2 shows that EP/DOPO+MMT has two characteristic peaks at 7.2° and 5.0°, respectively. This result shows that there were some montmorillonite samples that were able to retain the original structure of MMT and some montmorillonite samples with increased layer spacing. In other words, DOPO will intercalate with MMT in the physical mixing process of DOPO+MMT and EP. However, in the XRD pattern of EP/DOPO-MMT, the characteristic peaks of montmorillonite, such as those at 7.2° and 3.6°, disappeared. This result indicates that the montmorillonite in the EP/ DOPO-MMT samples mainly exists in the exfoliated or amorphous state. Therefore, the DOPO-MMT sample was well dispersed in EP/DOPO-MMT composites.

The possible reasons why MMT can achieve good dispersion in EP/ DOPO-MMT are analyzed as follows: The active hydrogen in DOPO can be easily introduced into the structure of EP through the reactions of the P-H bond with functional groups in the molecules of EP, such as the unsat C=C bond [32]. The epoxy molecules will therefore be dragged inside of the montmorillonite lamella. Finally, the interlaminates were extended and peeled into montmorillonite nanosheets.

### 3.3. SEM Analysis of Epoxy Resin Composite

**Figure 3:**
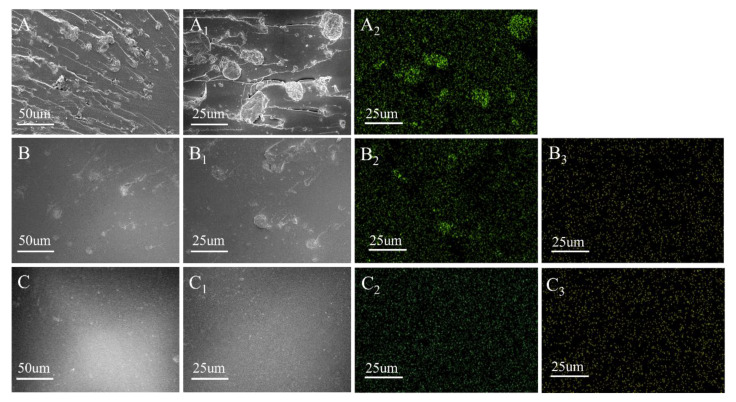
SEM diagram of EPs. (**A**,**A_1_**) EP/MMT; (**B**,**B_1_**) EP/DOPO+MMT; (**C**,**C_1_**) EP/DOPO-MMT; (**A_2_**,**B_2_**,**C_2_**)Si distribution mapping; (**B_3_**,**C_3_**)P distribution mapping.

The obtained EP/MMT, EP/DOPO+MMT, and EP/DOPO-MMT samples were characterized by SEM to study the dispersion state of MMT in the composite samples, as shown in Figure 3.

The dispersibility of montmorillonite in the epoxy resin composites can be visually seen from the SEM image in Figure 3. In Figure 3A, many agglomerated particles and pore defects are located near the particles and can be observed at the EP/MMT section. In Figure 3B, the aggregated particles can also be observed in the EP/DOPO+MMT section, and the size of the particles is slightly smaller than that in Figure 3A. In Figure 3C, EP/DOPO-MMT shows a smooth brittle section, and the montmorillonite particles that are of a microscopic size are not easily observed. The distribution of the Si and P elements on the surface of EP/MMT and EP/DOPO+MMT is shown in Figure 3A_2_,B_2_,C_2_,B_3_,C_3_, as displayed by SEM-EDX. The Si elements were unevenly distributed on the surface of EP/MMT and EP/DOPO+MMT, and the P elements were evenly distributed on the surface of EP/DOPO+MMT and EP/DOPO-MMT. This indicates that DOPO is uniformly dispersed in EP, and montmorillonite without DOPO modification were locally aggregated in EP; as such, the intercalated organic montmorillonite DOPO-MMT with larger d-spacing can be dispersed evenly in an epoxy resin matrix without obvious aggregation. The SEM and SEM-EDX results of the samples were consistent.

### 3.4. TEM Analysis of Epoxy Resin Composite

**Figure 4:**
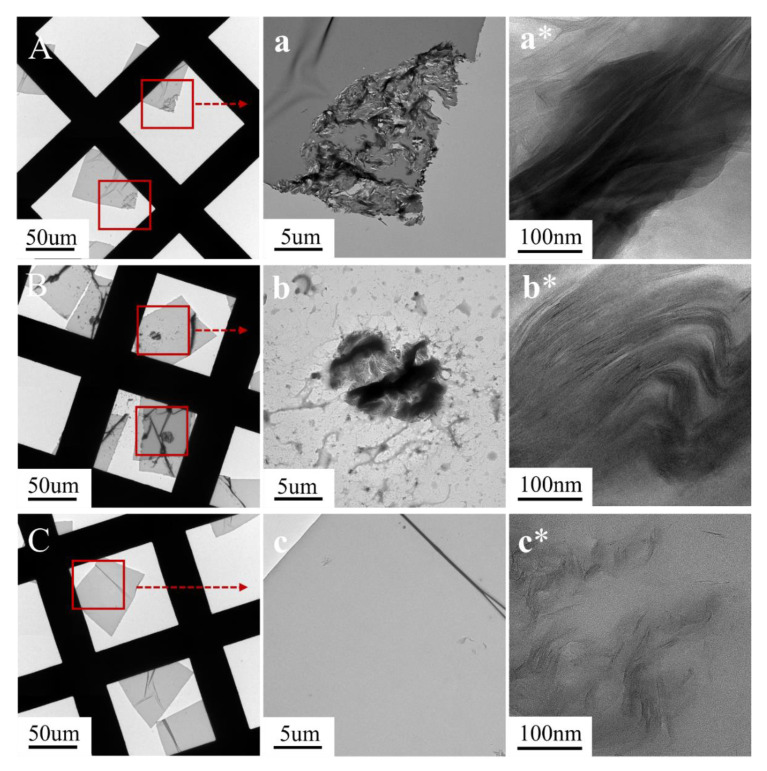
TEM image of EPs. (**A**,**a**,a*) EP/MMT; (**B**,**b**,b*); EP/DOPO+MMT; (**C**,**c**,c*) EP/DOPO-MMT.

The dispersion and morphology of MMT in the EP/MMT, EP/DOPO+MMT, and EP/DOPO-MMT samples were observed by TEM, as shown in Figure 4.

Figure 4 shows the TEM images of EP/MMT, EP/DOPO+MMT, and EP/DOPO-MMT with different magnifications. As it can be seen from Figure 4A,a,B,b,C,c, MMT still exists in the form of particles in EP/MMT and EP/DOPO+MMT composites, while almost no obvious particles can be seen in EP/DOPO-MMT. The results show that unmodified MMT and physically mixed DOPO+MMT cannot be uniformly dispersed in EP, and the results are consistent with the SEM results.

Meanwhile, it can be seen from Figure 4a*,b*,c* that the MMT lamellar layers were tightly packed in EP/MMT, and the lamellar spacing was small. This indicates that MMT was not intercalated or exfoliated and that MMT still exists in epoxy resin in the form of lamellar stack particles. In the EP/DOPO+MMT sample, the MMT lamellar spacing was larger in some parts than it was in EP/MMT. These results indicate that the intercalation of MMT occurred in these parts, but the lamellar structure was not separated and dispersed, which is consistent with the XRD results. The lamellar structure of MMT in EP/DOPO-MMT almost disappeared. Instead, MMT was exfoliated in the EP system in the form of multilayer nanosheets or monolayer sheets, and the lamellar spacing of the montmorillonite was roughly calculated in the range of 10~30 nm. The TEM results show that the montmorillonite lamellae in EP/DOPO-MMT were dispersed at the nanometer level.

### 3.5. LOI and UL-94 Test of Epoxy Resin Composites

LOI and UL-94 vertical burning tests are two typical small-scale evaluation methods for the combustion performance of polymer materials, and the results of the LOI and UL-94 vertical burning tests for epoxy resin composites are shown in Table 1.

As it can be seen from Table 1, the LOI of pure EP, EP/MMT, and EP/DOPO are 23.0%, 25.0%, and 32.6%, respectively. These results indicate that both DOPO and MMT can improve the LOI of epoxy resin and can contribute to the flame-retardant performance of epoxy resin. However, the contribution of DOPO is obviously better than that of MMT. According to the results of the LOI of EP/DOPO+MMT (32.1%) and EP/DOPO-MMT (32.9%), the contribution of the intercalated DOPO-MMT to the flame retardation of EP is higher than that of the physically mixed DOPO+MMT. The results show that the nano dispersed MMT contributed to the flame retardancy. Meanwhile, EP/DOPO-MMT also had a higher LOI than EP/DOPO. In other words, when 1/3 of the mixture was MMT instead of DOPO, the flame-retardant performance of EP/DOPO-MMT did not decrease, but it was higher than EP/DOPO or EP/MMT. The results show that the flame retardancy of MMT was lower than that of DOPO and that the synergistic effect of MMT and DOPO was the main reason for improving the flame retardancy of EP.

The same results were found for the UL-94 test as well. After being ignited for 10 s and after removing the ignition source, pure EP continued to burn with molten drip, and no char layer formed after testing. However, no droplet was observed in the EP/MMT sample during the test. The flame of EP/DOPO and EP/DOPO+MMT was self-extinguishing after burning for a certain period of time (t_1_ and t_2_ of EP/DOPO+MMT were longer than that of EP/DOPO). Their flame retardancy level reached V-1. The t_1_ and t_2_ of EP/ DOPO-MMT were 6 s and 9 s, respectively, and the flame-retardant performance was the best of all, reaching V-0. The results showed that DOPO and MMT have a synergistic flame-retardant effect on epoxy resin, and the flame-retardant effect of the intercalated DOPO-MMT is the best among that of DOPO and the physical mixture of DOPO+MMT. This indicates that EP/DOPO-MMT has excellent flame retardancy compared to the other samples, which is mainly due to the nano-scale dispersion of the montmorillonite and the synergistic effect of DOPO and MMT. In the case of an open flame, the DOPO in DOPO-MMT plays the role of the flame retardant and a role in carbon formation firstly, and then the inorganic nanosheet MMT assists the DOPO in preventing the material from generating flames and high-temperature diffusion, promoting carbon formation.

### 3.6. Thermal Stability of EPs

**Figure 5:**
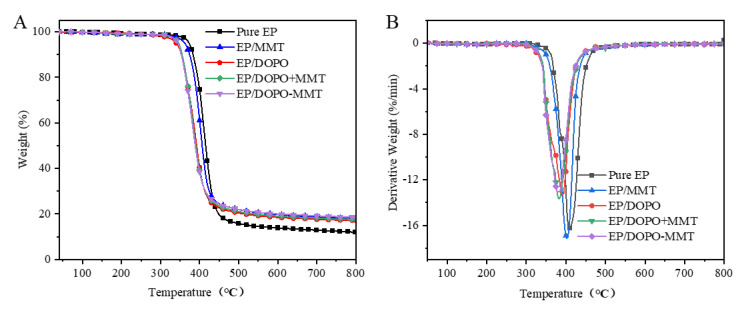
TG (**A**) and DTG (**B**) curves of EPs in N_2_.

TGA analysis results of epoxy resin composites in N_2_ are shown in Table 2 and Figure 5.

According to the results in Table 2 and Figure 5, the T_oneset_ and T_max_ of EP/MMT obtained after MMT was added to EP decreased by 9.1 °C and 5.9 °C compared to EP. When DOPO was added to EP, the T_oneset_ and T_max_ of the obtained EP/DOPO were significantly lower than those of EP, reaching 32.3 °C and 18.4 °C, respectively. These results indicate that the addition of MMT and DOPO can reduce the thermal stability of EP and can promote the advanced thermal decomposition of EP and that DOPO has a more significant effect. There are probably three main reasons for this phenomenon. First, the active hydrogen in DOPO can be easily introduced into the structure of EP through the reactions of the P–H bond with the functional groups in the molecules of EP, such as unsaturated C=C bonds; this affects the thermal stability of EP. Second, the thermal decomposition temperature of DOPO is lower than that of EP, which contributes negatively to the thermal stability of EP. Third, the acid sites on the surface of MMT stone have a catalytic effect on the thermal decomposition of EP, resulting in a slight decrease in thermal stability. The lower T_max_ for EP/DOPO-MMT compared to EP/DOPO+MMT further illustrates this point as well. The exfoliated degree of MMT in EP/DOPO-MMT was much higher than that of EP/DOPO+MMT, so the specific surface of MMT in EP/DOPO-MMT was greater than that of EP/DOPO+MMT. Therefore, the amount of acid sites of the MMT in EP/DOPO-MMT should be much higher than that of EP/DOPO+MMT, resulting in more thermal decomposition and a lower T_max_ for EP/DOPO-MMT. As it can be observed from thermogravimetric mass residual rate at 900 °C, the residual rate of EP/DOPO-MMT is 18.3%, which is the highest among the different EPs. The results show that montmorillonite was exfoliated and dispersed well in EP and that it can effectively promote the carbonization of epoxy resin. In addition, the mass residual rate of EP/MMT was 18.1%, which was slightly higher than that of EP/DOPO+MMT and EP/DOPO. One of the main reasons for this is the high MMT content in EP/MMT.

**Figure 6:**
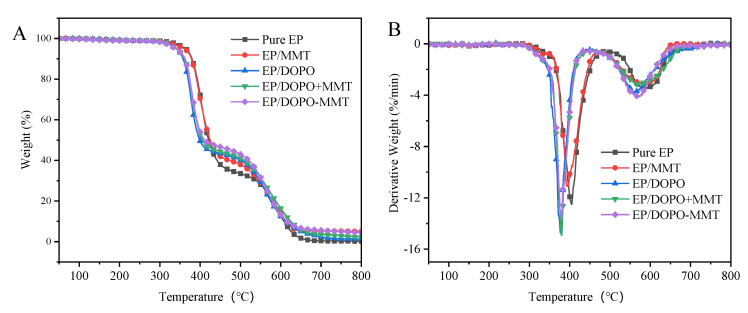
TG (**A**) and DTG (**B**) curves of EPs in air.

TGA analysis results of Eps in air are shown in Table 3 and Figure 6. It can be seen that different from one decomposition stage in the nitrogen atmosphere, the thermal decomposition of epoxy resin composites in air atmosphere presents two stages, i.e., 340~480 °C and 480~650 °C. The first stage was mainly the thermal decomposition of the epoxy resin matrix, and the second stage was the further oxidative decomposition of unstable carbon formed in the previous stage at a high temperature. The T_oneset_ and T_max_ 1 of EP/DOPO, EP/DOPO+MMT, and EP/DOPO-MMT were significantly lower than pure EP, indicating that the addition of DOPO could reduce the thermal stability of EP. The mass residue ratio of EP/DOPO-MMT at 900 °C was 3.19%, which was higher than that of EP/DOPO and EP/DOPO+MMT (1.09% and 0.43%, respectively), indicating that the DOPO-MMT nanocompounds had a better effect in terms of promoting carbon layer formation in EP than DOPO, MMT, and physically mixed DOPO+MMT.

### 3.7. Glass Transition of EPs

**Figure 7:**
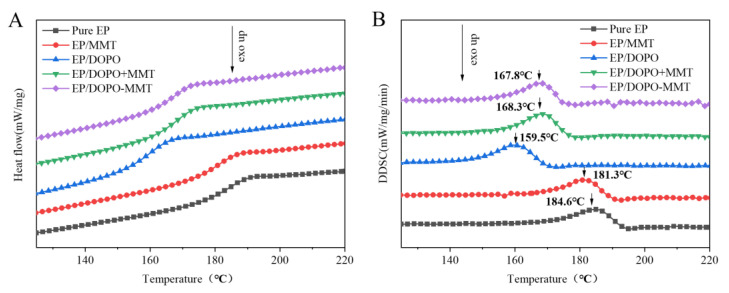
DSC (**A**) and DDSC (**B**) curves of EPs.

The glass transition temperature (Tg) of pure EP was the highest (184.6 °C), as shown in Figure 7. Compared to pure EP, the Tg of EP/DOPO decreased sharply (159.5 °C), which was mainly due to the plasticization of DOPO. The Tg of EP/MMT decreased slightly, indicating that the MMT with micron-sized particles had little effect on the glass transition temperature of epoxy resin. The EP/DOPO+MMT and EP/DOPO-MMT exhibited the medium Tg between the EP/MMT and EP/DOPO, and the Tg of EP/DOPO-MMT was slightly lower than that of EP/DOPO+MMT. The main reason for this was that the MMT that was intercalated by DOPO could have had good dispersion (intercalation and exfoliation) in the EP matrix, and it may have reduced the intermolecular force of EP.

### 3.8. CONE Test of Epoxy Resin Composites

**Figure 8:**
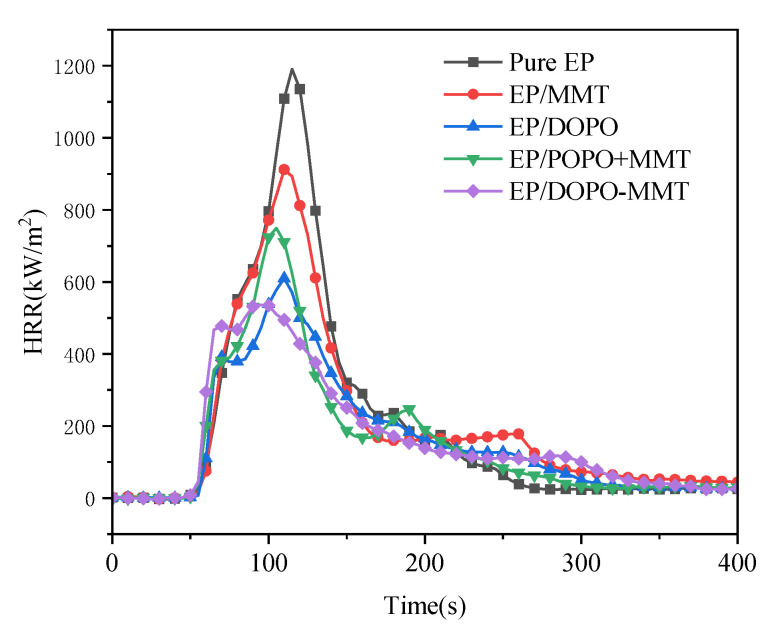
Heat release rate (HRR) curves of epoxy resin composites.

**Figure 9:**
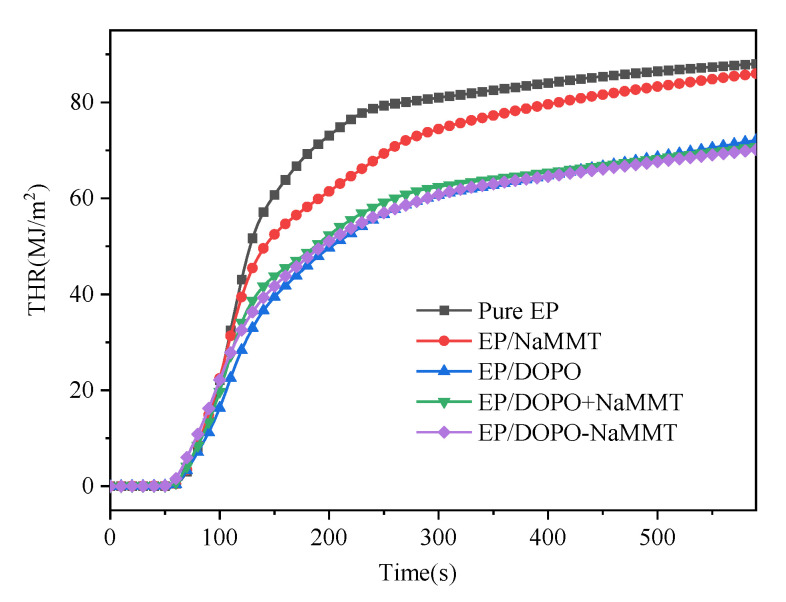
THR curves of epoxy resin composites.

The CONE test results of pure EP and four EP composites are shown in Table 4, Figure 8 and Figure 9.

As shown in Table 4, the TTI of pure EP and EP/MMT is 41 s, that of EP/DOPO is 53 s, and that of EP/DOPO+MMT and EP/ DOPO-MMT is 45 s. The results show that the addition of MMT had almost no effect on TTI, while the addition of DOPO had a positive effect on the increase of TTI, which possibly delayed the occurrence of combustion.

According to the PHRR data in Figure 8 and Table 4, the order of the PHRR of the five samples is: pure EP > EP/MMT > EP/DOPO+MMT > EP/DOPO > EP/DOPO-MMT. The p-HRR value (1190 kW/m^2^) of pure EP was the highest. With the addition of MMT and DOPO, the PHRR value of the EP composite samples decreased. In particular, the addition of DOPO-MMT resulted in the biggest reduction of EP/DOPO-MMT, and the PHRR value decreased to 563 kW/m^2^, 55.0% lower than that of pure EP, which was the lowest among all of the samples. It is also can be observed in Figure 8 that the HRR curves of EP/DOPO and EP/ DOPO-MMT both have two peaks. The first peak was formed by the ignition of the pyrolysis gas generated during the initial combustion. After the initial combustion, a char layer of a certain strength will be formed, which has significance in terms of preventing the rapid rise of HRR in the early stages of combustion. As thermal radiation progresses, the rupture of the char layer leads to secondary combustion, and formation of a second HRR peak. However, for the EP/DOPO+MMT with poor montmorillonite dispersion, it is difficult to form a stable char layer at the initial stage, resulting in a high value of the first p-HRR. Therefore, a good synergistic flame retardant effect between DOPO and MMT cannot be obtained using MMT without the modification of DOPO.

The THR of the modified EPs was significantly lower than that of pure EP, as shown in Table 4 and Figure 9. Compared to pure EP, the THR of EP/MMT, EP/DOPO, EP/DOPO+MMT, and EP/DOPO-MMT decreased by 2.3%, 18.1%, 19.7%, and 20.3%, respectively. This indicates that the THR of the epoxy resin composite could be reduced by using both DOPO and MMT, and the contribution of the DOPO-MMT nanocomposite to the THR reduction is slightly better than that of the DOPO+MMT mixture.

The semi-quantitative calculation of the synergistic effectivity (*SE*) can be calculated by Equation (1) [33]. It enables a clear differentiation between synergism, cooperation, and antagonism. In this calculation, we assume that the concentrations of the flame retardants DOPO and MMT are proportional to their respective Δ*PHRR*.

(1) $$\;SE=\frac{\left(∆PHRR_{ac}^{i}+∆PHRR_{ac}^{h}\right)}{\left(∆PHRR_{0}^{i}+∆PHRR_{0}^{h}\right)}$$

where “*i*” is the DOPO, and “*h*” is MMT. “*ac*” indicates the actual value of DPHRR in a given experiment in which both the FR agent and synergist are present. The value “0” indicates the calculated additive sum of Δ*PHRR* of both ingredients obtained in the experiments in which only one agent is used. The concentrations of the two ingredients are the same as they would be in the actual experiments in which both are included. The case in which there is no synergism or antagonism, called “cooperation”, is when the value of *SE* is 1. *SE* values above 1 indicate synergism, while those below 1 indicate antagonism. The result of the synergistic effectivity related to PHRR are shown in Table 5. These results indicate that the nano-dispersed MMT and DOPO have a flame-retardant synergistic effect in EP.

The values of TSR, ASEA, PCOP, and PCO_2_P are also shown in Table 4. Compared to pure EP and EP/DOPO, the TSR and ASEA values of EP/MMT were significantly decreased, indicating that MMT could inhibit the generation and release of smoke. The TSR and ASEA values of EP/DOPO-MMT were the lowest among all of the Eps, indicating that the nano-dispersed MMT had a more obvious effect on inhibiting the generation and release of smoke. Moreover, the addition of the DOPO-MMT nanocomposite reduced the CO release rate but did not in-crease the CO_2_ release rate at the same time. These results indicate that the effect of the DOPO-MMT nanocomposite in promoting the char formation of epoxy resin is more obvious than that of DOPO and the physical mixture of DOPO + MMT.

### 3.9. Analysis of Condensed Phase of Epoxy Resin Composites CONE of Epoxy Resin Composites

Photographs of the sample after the conical calorimetry test are shown in Figure 8.

**Figure 10:**
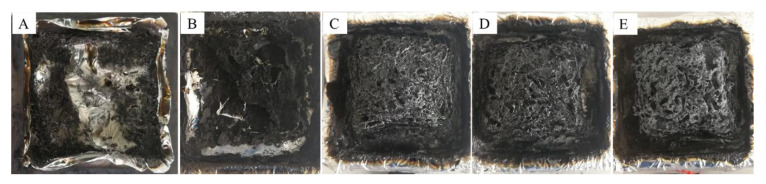
Char residue of epoxy resin composite. (**A**) Pure EP; (**B**) EP/MMT; (**C**) EP/DOPO; (**D**) EP/DOPO+MMT; (**E**) EP/DOPO-MMT.

As shown in Figure 10, pure EP could not form a char layer (Figure 10A). The epoxy resin with MMT also failed to form a complete char layer (Figure 10B), but some fluffy residues were observed that had a floccule structure and that played a limited role in improving the flame-retardant properties of the epoxy resin. The epoxy resin composites with DOPO, the physical mixture of DOPO + MMT, and the DOPO-MMT nanocomposite were able to form an expanded intact char layer. The residue of EP/DOPO-MMT had a unique morphology, showing a continuous white carbon layer with a bulging outer layer. The partial white material on the surface should be the outer char layer, and it should have a high silicon content produced by the combustion of the nano-dispersed montmorillonite in the epoxy resin. The char layer can effectively protect the internal epoxy resin matrix. The EP/DOPO and EP/DOPO+MMT char residue samples were compared as well. The synergistic effect of DOPO and MMT promoted the formation of EP/DOPO-MMT char residue with a unique morphology.

**Figure 11:**
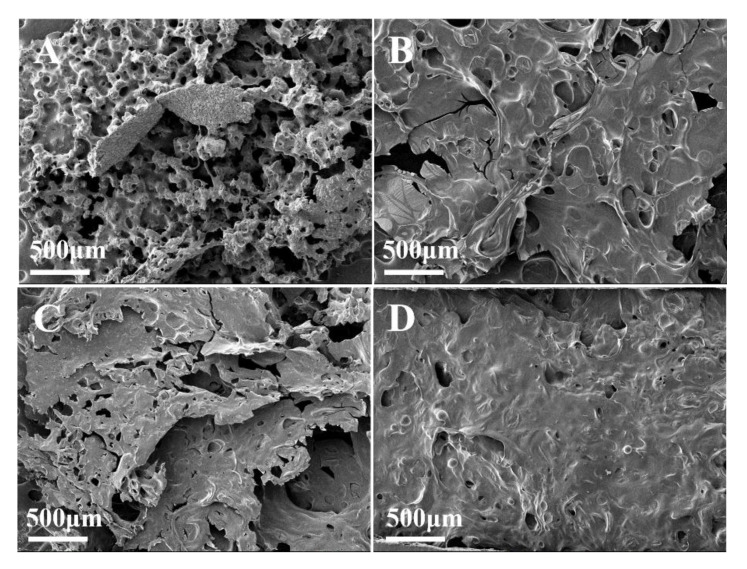
SEM image of char layer of epoxy resin composite. (**A**) EP/MMT, (**B**) EP/DOPO, (**C**) EP/DOPO+MMT, (**D**) EP/DOPO-MMT.

In Figure 11A, EP/MMT shows a barely formed char layer, which consists of many small coarse particles and deep pores of different sizes. In Figure 11B, EP/DOPO formed a relatively flat char layer structure with holes in the carbon layer. In Figure 11C, EP/DOPO+MMT formed a coarser char layer with large pores. Compared to the previous three samples, the internal char residue of EP/DOPO-MMT in Figure 11D is thick and smooth, and the char layer is more continuous and compact. These results indicate that EP/DOPO-MMT has a more effective char layer structure, which results in the best flame-retardant performance.

## 4. Conclusions

In this article, intercalated organic montmorillonite DOPO-MMT was prepared by melting and modifying MMT with phosphorous flame-retardant DOPO. Compared to the preparation of DOPO-MMT using the solution-ethanol method, the DOPO-MMT discussed in this article was prepared without ethanol solvent, with less flame retardant, and by means of a simple environmentally friendly and low production cost process. In the EP/DOPO-MMT composite, the DOPO-MMT was evenly dispersed in the EP in the form of nanosheets. In the test of the flame retardancy of the epoxy resin composites, the contribution of DOPO-MMT to the EP flame retardancy was the best compared to physically mixed DOPO+MMT, DOPO, and MMT. The EP/DOPO-MMT composite also reached the V-0 grade. This is because that the intercalated DOPO-MMT was nano-dispersed in the epoxy resin and because of the synergistic effect between DOPO and MMT. At the same time, the preparation technology used for the intercalated organic montmorillonite DOPO-MMT, which was achieved through melting method in this article, provides a new way for the preparation of modified montmorillonite a with phosphorus-based flame retardant. Additionally, the prepared EP/DOPO-MMT composites can be widely used in printed circuit boards, electronic packaging, aerospace, and other fields requiring high flame retardant performance

## Figures and Tables

**Table 1:** Flame-retardant properties of EPs.

Samples	LOI (%)	UL-94			
		t_1_ (s)	t_2_ (s)	Dripping	Rating
Pure EP	23.0	>60	/	YES	NR
EP/MMT	25.0	>60	/	NO	NR
EP/DOPO	32.6	7	17	NO	V-1
EP/DOPO+MMT	32.1	16	22	NO	V-1
EP/DOPO-MMT	32.9	6	9	NO	V-0

**Table 2:** Thermogravimetric data of Eps in N_2_.

Samples	T_oneset_ (°C)	T_max_ (°C)	Maximum Thermal Weight Loss Rate (%/min)	Mass Residue Ratio at 900 °C (%)
Pure EP	372.9	408.5	−16.31	9.4
EP/MMT	362.0	402.6	−17.11	18.1
EP/DOPO	340.6	390.1	−13.3	17.0
EP/DOPO+MMT	346.4	382.4	−13.63	17.5
EP/DOPO-MMT	344.8	380.0	−13.07	18.3

**Table 3:** Thermogravimetric data of Eps in air.

Samples	T_oneset_ (°C)	T_max_ 1 (°C)	T_max_ 2 (°C)	Mass Residue Ratio at 900 °C (%)
Pure EP	363.8	404.8	580.5	0
EP/MMT	360.2	401.3	581.0	3.88
EP/DOPO	339.6	377.0	560.5	0.43
EP/DOPO+MMT	341.1	369.8	580.0	1.09
EP/DOPO-MMT	338.9	369.6	572.2	3.19

**Table 4:** CONE data of epoxy resin composites.

Samples	TTI (s)	PHRR (kW/m^2^)	THR (MJ/m^2^)	TSR (m^2^/m^2^)	ASEA (m^2^/kg)	PCOP (g/s)	PCO_2_P (g/s)
Pure EP	41	1190	88.0	3658	843	0.030	0.615
EP/MMT	41	911	86.0	3396	691	0.029	0.564
EP/DOPO	53	610	72.1	3792	867	0.035	0.368
EP/DOPO+MMT	45	749	70.7	3533	705	0.037	0.449
EP/DOPO-MMT	45	536	70.1	3346	668	0.026	0.361

TTI: ignition time, PHRR: peak of heat release rate, THR: total heat release, TSR: total smoke release, ASEA: average specific extinction area.

**Table 5:** Synergistic effectivity related to PHRR.

Samples	Contents	ΔPHRR (kW/m^2^)	SE
EP/MMT	6% MMT	279	-
EP/DOPO	6% DOPO	580	-
EP/DOPO+MMT	6% DOPO+MMT (4% DOPO 2%MMT)	441	0.92
EP/DOPO-MMT	6% DOPO-MMT (4% DOPO 2%MMT)	654	1.36

## Data Availability

The data presented in this study are available upon request from the corresponding author.
